# Hypoxia-Inducible Factors Signaling in Osteogenesis and Skeletal Repair

**DOI:** 10.3390/ijms231911201

**Published:** 2022-09-23

**Authors:** Qiuyue Qin, Yiping Liu, Zhen Yang, Maierhaba Aimaijiang, Rui Ma, Yixin Yang, Yidi Zhang, Yanmin Zhou

**Affiliations:** Department of Oral Implantology, Hospital of Stomatology, Jilin University, Changchun 130021, China

**Keywords:** hypoxia-inducible factors, osteogenesis, vascular endothelial growth factors, erythropoietin, wnt

## Abstract

Sufficient oxygen is required to maintain normal cellular and physiological function, such as a creature’s development, breeding, and homeostasis. Lately, some researchers have reported that both pathological hypoxia and environmental hypoxia might affect bone health. Adaptation to hypoxia is a pivotal cellular event in normal cell development and differentiation and in pathological settings such as ischemia. As central mediators of homeostasis, hypoxia-inducible transcription factors (HIFs) can allow cells to survive in a low-oxygen environment and are essential for the regulation of osteogenesis and skeletal repair. From this perspective, we summarized the role of HIF-1 and HIF-2 in signaling pathways implicated in bone development and skeletal repair and outlined the molecular mechanism of regulation of downstream growth factors and protein molecules such as VEGF, EPO, and so on. All of these present an opportunity for developing therapies for bone regeneration.

## 1. Introduction

If the molecular properties of intracellular signals are different, the type of signals, the pathways included, and subsequent regulatory interactions will vary, which will affect the final cell activity. Osteogenesis and bone regeneration have been complex systems for analyzing interrelated signals and studying the mechanisms. A clear understanding of these mechanisms could help regulate bone mass through mediating cytokines or altering the cell growth environment.

In the physiological state, osseous tissues are formed via two different methods: endochondral ossification or intramembranous ossification. Endochondral ossification requires a cartilage model and is replaced by bone, while mesenchymal condensations lead directly to intramembranous ossification. The processes of endochondral ossification include formation of the mesenchymal condensation, chondrocyte differentiation and maturation, and osteoblast development and mineralization [1] (Long and Ornitz, 2013). Mesenchymal cells condense at the site of the future cartilage template and then differentiate into chondrocytes. Chondrocytes proliferate and some differentiate into hypertrophic chondrocytes. The center of the cartilage mold becomes increasingly hypoxic, and these cells secrete angiogenic stimuli, followed by vascular invasion. Osteoblast precursors and osteoclasts also enter the hypertrophic cartilage area and erode the cartilage. Osteoblasts secrete type I collagen to form the bone matrix, followed by the primary ossification center. Postnatally, the secondary ossification centers form. The invasion of blood vessels into hypertrophic cartilage is a key step. The blood vessels bring mesenchymal cells into the front of mineralization and then differentiate into osteoblasts to participate in osteogenesis [2]. Most bones are formed by endochondral ossification, including long bones such as the femur and tibia, while flat bones, such as the skull, form by intramembranous ossification [3]. Many factors, including inflammation, tumors, and trauma, could cause bone defects, decreasing the quality of life [4]. The healing of bone defects is a complex and continuous process in which many cells constantly coordinate. Bone regeneration and remodeling consist of seven sequential phases: quiescence, activation, resorption, reversal, activation, mineralization, and termination. This process is regulated by a variety of factors, including calcitonin, parathyroid hormone, vitamin D3 [1,25(OH)_2_ vitamin D3]. In addition to systemic hormones, growth factors such as insulin-like growth factors (IGFs), transforming growth factors (TGF)-β, fibroblast growth factors (FGFs), epidermal growth factors (EGF), Wnts, and bone morphogenetic proteins (BMPs) play important roles in physiological bone remodeling. BMPs mainly mediate the Smad and MAPK pathways to increase Runx2 and Osx expression, which are essential for bone remodeling and maintenance of bone mass. TGF-β has double effects on osteoblasts, stimulating the differentiation of early osteoblasts and secreting BMPs while inhibiting the differentiation of late osteoblasts. FGF23, an important member of the FGF family, is mainly produced by osteocytes and osteoblasts and directly stimulates the differentiation of BMSCs through relevant physiological pathways related to phosphate homeostasis. IGF-1 promotes the proliferation and function of osteoblasts to maintain skeletal homeostasis. Wnt signaling has a direct and indirect effect on bone cell lineages, leading to an overall increase in bone formation [5] (Siddiqui and Partridge, 2016). Usually, multiple factors, including external elements, collaborate to form new mineralized tissues and increase bone volume [6]. At present, bone regeneration is still a challenge faced in bone surgery.

As a signal molecule, oxygen can regulate cellular survival, metabolism, migration, differentiation, and cell-to-cell interactions. Consequently, it is significant to the wellbeing of all multicellular organisms. The structure of bone is relatively complicated, and the degree of oxygen in the central bone marrow is lower than that in the atmosphere. The arterial oxygen tension (PaO_2_) of normal adults is 80–100 mmHg, and the mixed venous oxygen tension (PVO_2_) is 35–45 mmHg. Researchers measured absolute local oxygen tension (pO_2_) in the bone marrow (BM), which is significantly low (<32 mmHg). It is lower than the local pO_2_ in the periosteum, cortical bone, and brain microvessels [7]. In addition, research found that the local pO_2_ in heterogeneities and pO_2_ in deeper peri-sinusoidal regions are quite low (~9.9 mmHg, or 1.3%), which may be due to higher cell density consuming more oxygen despite dense vascularity [8]. A certain degree of hypoxia in bone marrow can stimulate angiogenesis, increase the ability of blood to carry oxygen, and change cell metabolism from aerobic to anaerobic respiration [9]. Osteocytes, such as mid-cortical osteocytes, are more resistant to ischemia-induced stress in the hypoxic environment [10]. Hypoxia also affects self-renewal, proliferation, stem-cell differentiation, and other physiological aspects. Under the condition of hypoxia, the cell and nuclear morphologies in hMSCs show differences, and the formation and organization of extracellular matrix (ECM) proteins are enhanced, while the growth rates of hMSCs are maintained [11]. In the process of osteogenesis, hypoxia promotes angiogenesis, and the underlying mechanisms may involve autophagy, VEGF, the Wnt/β-catenin signaling pathway, and so on [12]. In addition, when bone tissue is injured, blood vessels in the periosteum, bone, medullary cavity, and adjacent soft tissues rupture and hemorrhage, forming hematoma at the fracture site, which restricts blood perfusion to the fracture site. As a result, blood flow drops immediately, leading to regional hypoxia. However, in part, hypoxia is also involved in promoting fracture healing [13]. Therefore, hypoxia plays a particular role in bone homeostasis under physiological and pathological aspects, and exploring and clarifying the underlying mechanism is necessary. Under the hypoxic condition, cells activate the hypoxia-inducible factor (HIF)-mediated survival mechanism to regulate proliferation activity and function and prevent cell death caused by hypoxia [14].

## 2. Hypoxia-Inducible Factors

Hypoxia-inducible factors (HIFs) are transcriptional activator complexes that perform a central role in the expression of oxygen-regulated genes. These genes are involved in the proliferation and apoptosis of cells, angiogenesis, erythropoiesis, energy metabolism, vasomotor function, and so on [15,16]. Thus, HIFs are essential for normal growth and development and also participate in the pathological processes, including tumor progression and tissue regeneration [17]. Heterodimeric transcription factors (HIFs) complex are composed of α-subunits (HIF-1α, HIF-2α, and HIF-3α) and the β-subunit (HIF-1β)/aryl hydrocarbon receptor nuclear translocator (ARNT). HIF-1β/ARNT is expressed stably in cells, whereas HIF-αs are degraded under the condition of normal oxygen bioavailability and accumulate rapidly in a hypoxic environment. HIF-1α, HIF-2α, and HIF-3α bind to HIF-1β to form HIF-1, HIF-2, and HIF-3, respectively. Thus, the stability of the HIF-1α subunit seems to determine HIF-1 formation. Similarly, the formation of HIF-2 is mainly determined by the abundance of the HIF-2α subunit.

**Figure 1:**
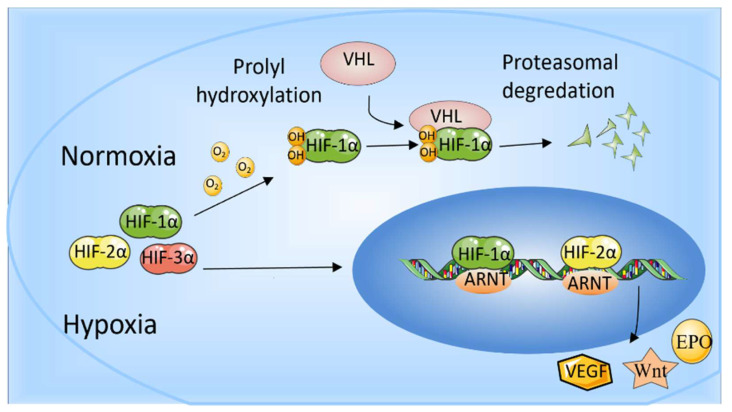
The role of HIF-1α and HIF-2α. When oxygen levels are low (hypoxia), HIF-1α and HIF-2α are protected from degradation and accumulate in the nucleus, where they bind to ARNT and bind to specific DNA fragments in hypoxia regulatory genes. At normal oxygen levels, oxygen regulates the degradation process by adding hydroxyl groups to HIF-αs. VHL recognizes and forms a complex that carries HIF-αs and degrades them in an oxygen-dependent manner.

In mammalian cells, three HIF-α subunit isoforms (HIF-1α, HIF-2α, and HIF-3α) are encoded by three HIF-α genes: HIF1A, HIF2A, and HIF3A, respectively. When oxygen concentration drops to <5%, HIF-1α is stably expressed, enters the nucleus, dimerizes with HIF-1β, and binds to HIF-response elements (HRE) of targeted gene promoters [16]. When oxygen is abundant in cells (>5%), the Prolyl-4-hydroxylases (PHDs) bind to HIF-1α and hydroxylate the proline residues, which leads to the recruitment of the Von Hippel-Landau (VHL) tumor suppressor E3 ligase complex. Eventually, the proteasomal is poly-ubiquitylated and degraded [18]. In addition, factor inhibiting HIF (FIH) also restricts the binding of HIF-αs to transcriptional co-activators CBP/p300 through hydroxylating (N-terminal) asparaginyl residues when oxygen is abundant [19]. HIF-2α is regulated by oxygen in a similar manner to HIF-1α. In addition to intracellular oxygen tension, several growth factors can also regulate HIF-α subunits in a hypoxia-independent way [20]. HIF-1 has been studied more extensively than HIF-2, and HIF-1 and HIF-2 have overlapping and unique biological functions. It is reported that HIF-1α responds to acute hypoxia mainly, whereas HIF-2α is the prime subunit that responded to chronic exposure to low oxygen at high altitudes [21]. HIF-1α is generally expressed in cells and regulates downstream genes, including VEGF, GLUT-1, AK-3, ALD-A, PGK-1, PFK-L, and LDH-A through binding to HRE to regulate many metabolic enzymes [22] (Maxwell, 1999). The role of HIF-1 in promoting angiogenesis also benefits cancer development. HIF-2 regulates erythropoiesis and vascularization and is essential for embryonic development [18]. In addition, HIF-2 is also involved in the progression and metastasis of solid tumors [23]. Another HIF-α protein, HIF-3α, can bind to ARNTs to restrain HIF-1α- or HIF-2α-mediated transcription, but its transcriptional capacity is weaker than other HIFs [16,24]. HIF-3α is relatively unknown in terms of regulating the hypoxia response, and many studies have shown that HIF-3α may play a dual part as a hypoxia-inducible transcription factor in recent years [25]. The determination of genome-wide binding of the human HIF-3 and its role requires extensive scientific research (Figure 1).

### Effect of HIFs on Bone

More recent studies have demonstrated the role of HIF-1 in bone growth and repair. Ref. [26] used spongy scaffolds that contained dimethyloxalylglycine (DMOG) in rat calvarial defects to imitate hypoxia to up-regulate HIF-1α, and found that angiogenesis was accelerated and bone regeneration was enhanced. Ref. [27] found that HIF-1α could facilitate osseointegration of tissue-engineered bone, dental implants, and new bone formation around implants, which was verified in a canine model. Another study has shown that expression of gingival HIF-1α protein in mice was apparently increased, and the ability of bone regeneration was enhanced at the onset of periodontitis resolution, after subcutaneous injection of 1,4-dihydrophenonthrolin-4-one-3-carboxylic acid (1, 4-DPCA/hydrogel), a hydrogel-formulated PHD inhibitor [28]. Gene ablation of *phd2* in chondrocytes promotes endochondral osteogenesis through up-regulation of HIF-1α signaling, resulting in a significant growth of long bones and vertebrae [29]. In the process of bone regeneration, HIF-1α not only promotes angiogenesis but also regulates metabolic adaptations by inducing glycolysis transformation to promote cell survival [30]. Hence, HIF-1 serves an indispensable role in osteogenesis and bone restoration [26,31]. When osteoblasts and other associated cells sense reduced oxygen tension, intracellular HIF-1α is stably expressed to regulate the expression of the angiogenic and osteogenic genes [32]. Additionally, the mechanisms by which HIF-1 regulates downstream genes to promote osteogenesis and bone repair are quite complex. The role of HIF-1 in mediating downstream signaling to regulate bone mass in different animal models or cells is displayed in Table 1.

The role of HIF-2 in osteogenesis is less understood compared to HIF-1 [42]. HIF-1 and HIF-2 have overlapping and opposite effects on the regulation of bone formation. Studies have suggested that HIF-2α can up-regulate the expression of VEGFA, COL10A1, and MMP13, and is a central transactivator of some key genes for endochondral ossification. When HIF-2α expression is reduced, chondrocyte hypertrophy, matrix degradation and vascularization, and other subsequent steps are impaired. Additionally, HIF-2α plays a more critical role in pathological endochondral ossification than in physiological endochondral ossification [43]. Studies have also shown that HIF-2 could up-regulate the expression of Sox9 to affect the differentiation of osteoblasts and regulate osteogenesis negatively, target Twist2 to down-regulate Runt-related transcription factor 2 (Runx2) and osteocalcin, and inhibit osteoblastic differentiation [44,45]. Moreover, HIF-2 might mediate the crosstalk between osteoblasts and osteoclasts by targeting RANKL in osteoprogenitor cells [44,46]. The up-regulation of HIF-2α expression in osteoblasts and osteoclasts is a novel intrinsic mediator of age-related bone loss [47]. In addition, studies have shown that HIF-2α, as a direct transcriptional target of NF-κB, destroys cartilage by regulating key catabolic genes in osteoarthritis (OA) [48,49]. However, HIF-1α inhibits the NF-κB-HIF-2α pathway to prevent cartilage degradation [50]. Bouaziz et al. demonstrated that HIF-1α interacts with β-catenin, which inhibits transcription factor 4-β-catenin transcriptional activity, suggesting that HIF-1α plays an important role in articular cartilage homeostasis and growth plate chondrocytes [51]. In the context of angiogenesis, HIF has been reported to control further up-regulation of hypoxia miR-424 in endothelial cells (ECs). This, in turn, contributes to HIF protein stabilization to adapt to low oxygen conditions and induces angiogenesis [52,53]. The role of HIF-2 in regulating bone homeostasis by mediating downstream signals in different animal models or cells is shown in Table 2.

The role of HIF signaling pathways implicated in osteogenesis and skeletal repair still has not been expatiated systematically. In this review, we highlight the recent studies on how cells and molecules play a role during skeletal development and repair through the HIF signaling pathway. To our knowledge, this review is the first to emphasize how the transcription factors HIF-1 and HIF-2, and their target genes regulate osteogenic process in response to hypoxia systematically. All of these present an opportunity for developing therapies for the regulation of bone regeneration and repair.

## 3. HIF/VEGF Pathways

**Figure 2:**
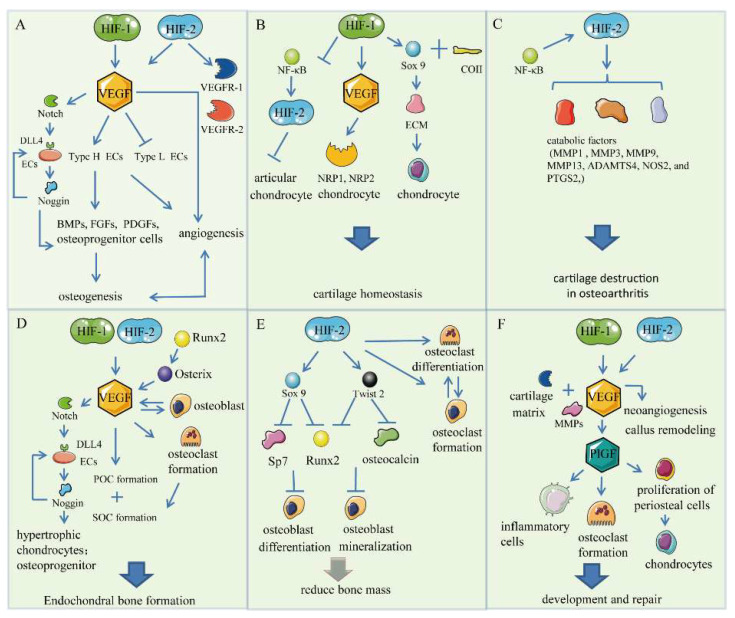
HIF/VEGF pathway. (**A**) Bone angiogenesis. VEGF is mediated by HIF-1 and HIF-2 to bone-specific ECs, type H, to promote bone angiogenesis. VEGF–Notch–Noggin pathway underscores the coupling that exists between angiogenesis and osteogenesis. (**B**) Chondrogenesis. VEGF binds to its receptors, NRP1 and NRP2, on the surface of chondrocytes. HIF-1 up-regulates Sox-9 and modifies COII after translation to promote cartilage homeostasis. (**C**) HIF-2 directly regulates catabolic factors to destroy cartilage in osteoarthritis. (**D**) Endochondral bone formation. HIF-1 and HIF-2 stimulate the expression of VEGF to promote the formation of the POCs and SOCs and mediate endochondral bone formation. (**E**) Regulation of bone mass by HIF-2. HIF-2 inhibits osteoblastic differentiation and mineralization. (**F**) Skeletal repair. HIF-1–VEGF–PlGF pathway plays a significant role in the process of fracture repair.

Vascular endothelial growth factor (VEGF) is a recognized and classic target of HIFs currently and is directly regulated by HIF-1 and HIF-2. HIF/VEGF pathways play an important role in skeletal and cartilage development as well as fracture repair. As a homodimeric glycoprotein with a size of 45 kDa, VEGF is a member of the dimeric cystine-knot growth factor superfamily, which mainly consists of VEGF-A (VEGF), VEGF-B, VEGF-C, VEGF-D, VEGF-E, and placental growth factor (PlGF) [6,59] (Hu and Olsen, 2016; Maes et al., 2012). VEGF mainly activates the angiogenic process and plays a role in physiological activities such as embryogenesis, skeletal growth, and reproductive functions, as well as in pathological activities such as blood vessel growth in tumors [60] (Ferrara et al., 2003). The primary target cells of VEGF are epidermal cells (ECs), but certain non-ECs such as osteoblasts have also been reported [61] (Watson and Adams, 2018). For instance, it is reported that VEGF participates in the regulation of osteoblast differentiation in the early stages of bone development [62,63] (Duan et al., 2015; Zavan et al., 2017). Additionally, VEGF is also expressed in other types of bone cells [14] (Stegen and Carmeliet, 2018). Researchers used VEGF-loaded scaffolds to investigate their role in promoting angiogenesis and bone regeneration in vivo [64] (Garcia et al., 2016). The regulation of VEGF by HIFs has been manifested in the following aspects of holding bone mass (Figure 2).

### 3.1. Bone Angiogenesis

HIF-1α can activate the transcription and expression of the VEGF gene and promote angiogenesis, which has been confirmed in several studies [26,65,66,67]. For example, Chen et al. demonstrated activated HIF-1α up-regulates VEGF-A, leading to a significant augmentation of functional vessels in the Matrigel plug angiogenesis assay [68]. The application of a hypoxia mimetic agent, deferoxamine (DFO), can activate the HIF-1/VEGF pathway to promote angiogenesis in osteonecrosis of the femoral head [69]. The underlying mechanisms of angiogenesis are as follows.

VEGF is activated by HIF-1α to recognize bone-specific ECs, in order to promote bone angiogenesis. Bones are rich in blood vessels, and the skeletal vascular bed is complex and contains different capillary subtypes that express various angiogenic factors [14,61,70]. According to the expression levels of the cell-surface markers endomucin (Emcn) and CD31, ECs can be divided into two subtypes, type H (CD31 ^hi^ Emcn ^hi^) and type L (CD31 ^lo^ Emcn ^lo^) [71]. Arterial blood flows through type-H capillaries to type-L capillaries, which branch from the metaphysis into the bone marrow in the diaphysis and then drain into the central vein [72]. Type-H vessels are beneficial to bone development because they can transport oxygen, nutrients, growth factors, and osteoprogenitor cells needed for bone growth to the sites of developing bone and promote bone formation. Compared with type-L vessels, type-H vessels are associated with osteoprogenitors expressing osterix and express high levels of pro-osteogenic factors, for instance, bone morphogenetic proteins (BMPs), fibroblast growth factors (FGFs), and platelet-derived growth factors (PDGFs) [61]. Hypoxia promotes the stabilization of HIF-1α in ECs, which increases the number of type-H vessels and osterix-expressing cells, leading to an increase in trabecular bone mass [4]. In conclusion, hypoxia can promote the formation of type-H vessels, stimulate the growth of the bone vascular system, and create a suitable molecular microenvironment for bone formation, which stimulates the differentiation of osteoprogenitors, coupling angiogenesis to osteogenesis [3]. The age-dependent decrease of type-H endothelium is also correlated with decreased HIF-1α, and the expression of HIF-1α may be related to age [73]. In turn, the expression of type-H and type-L vessels may also impact the expression of HIF-1α [73].

It has been reported that the VEGF–Notch–Noggin pathway plays an indispensable role in the coupling between angiogenesis and osteogenesis [74]. Notch signaling also promotes the release of Noggin, an angiocrine factor that antagonizes BMPs. It can stimulate the differentiation of osteoprogenitors and the maturation of chondrocytes, and ultimately regulate growth plate morphology and trabecular bone mass. At the same time, Noggin stimulates hypertrophic chondrocytes to secrete VEGF [75]. Increased vascularization may recruit amounts of osteogenic cells and osteoblasts that secrete osteogenic growth factors, such as BMP, that contribute to the maturation and differentiation of osteoblast and bone formation. Maturating osteoblasts also produce angiogenic factors such as PDGF and VEGF. It follows that there is a molecular crosstalk between endotheliocyte and osteoblastic cells because bone blood vessel growth and osteogenesis are coupled. In addition, Yang et al. found that endothelial Notch and HIF-1α activity are associated with the formation of type-H ECs [76]. Indeed, endothelial-cell-specific Notch activity accelerates the proliferation of bone ECs in the columns of the type H vessels [75]. All in all, HIF-1α may promote angiogenesis and osteogenesis by induction of type-H endothelium, regulating VEGF, and activating the VEGF–Notch–Noggin pathway (Figure 2A). Studies have suggested that HIF-2 also up-regulates VEGF and its receptors VEGFR-1 (Flt-1) and VEGFR-2 (Flk-1) by binding to HREs under hypoxia conditions, and is involved in several processes of angiogenesis, including cell proliferation, migration of blood vessels, and metastasis [77,78].

### 3.2. Chondrogenesis

As the epiphyseal growth plate expands, oxygen and nutrients are gradually deficient for the central chondrocytes, which activate HIF signaling to maintain the hypoxic chondrocytes’ survival and metabolism [14]. The HIFs/VEGF pathway conjointly governs the survival of hypoxic cartilage. As has been reported, VEGF is a critical component of the signaling pathway for chondrocyte survival. In addition to the role of angiogenesis, the important function of VEGF activated by HIF-1 is to support chondrocyte survival in epiphyseal cartilage [79]. Additionally, researchers believe that HIF-1, rather than HIF-2, is essential for growth plate cartilage development and that HIF-1 and HIF-2 in chondrocytes may play an antagonistic role [48,54]. When HIF-1α activity is inhibited in chondrocytes, cells die in the central regions of skeletal elements, as in VEGF-deficient bones [80]. Of note, some chondrocytes can be rescued from death by overexpression of VEGF in HIF-1α-deficient mice in the same way, indicating that HIF-1α is relevant to VEGF in support of chondrocyte survival [54]. Interestingly, while VEGF expression in hypertrophic chondrocytes does not appear to be HIF-1α-dependent, moderate (and later) VEGF expression in epiphyseal cells is regulated by HIF-1α [80]. Therefore, there may be two mechanisms to regulate the expression of VEGF in the growth plate: HIF-1α-dependent and HIF-1α-independent mechanisms. Identifying the receptors of VEGF on chondrocytes is essential to determine whether VEGF regulates chondrocytes directly and to understand the mechanisms of action of the HIF-1/VEGF pathway. VEGFR-2, as the main signal receptor of VEGF, is mainly expressed in ECs to mediate angiogenesis [60]. NRP1 and NRP2 are also VEGF receptors, mediating the actions of VEGF in chondrocytes. NRP1 has been reported to enhance the binding of VEGF to VEGFR-2 when NRP1 is expressed with VEGFR-2 simultaneously in cells. Further to this, Bmal1, as a core circadian rhythm gene, has also been found to regulate the HIF-1/VEGF pathway to influence cartilage development. Additionally, the ablation of Bmal1 reduces the expression of HIF-1α and VEGF, leading to decreased proliferation and increased apoptosis of chondrocytes [81]. In addition, HIF-1α activity directly promotes cartilage-specific ECM formation by stimulating the expression of Sox-9 and modifying collagen type II [82]. HIF-2 regulates catabolism in cartilage destruction. Studies have suggested that HIF-2 directly regulates catabolic factors such as MMP1, MMP3, MMP9, MMP13, ADAMTS4, NOS2, and PTGS2 to destroy cartilage in osteoarthritis, while the application of the specificity protein 1 (SP1) inhibitor Mithramycin A (MitA) could reduce the expression of HIF-2, thereby reducing the destruction of cartilage in osteoarthritis [48,83] have shown that HIF-1α suppresses the NF-κB-HIF-2α axis to regulate the growth of articular cartilage [84]. Taken together, the activated HIF-1α/VEGF pathway positively regulates epiphyseal cartilage. Additionally, HIF-1α can directly regulate cartilage-specific ECM, while HIF-2α has the opposite regulation effect (Figure 2B,C).

### 3.3. Endochondral Bone Formation

The process of endochondral bone formation usually includes four stages: the degeneration of cartilage and formation of the primary ossification centers (POCs), the construction of the bone marrow cavity, the formation of secondary ossification centers (SOCs), and the formation and remodeling of compact bone substance and diaphysis. It has been demonstrated that HIF signaling is indispensable in regulating chondrocyte differentiation during endochondral ossification. PHD2-HIF signaling regulates the maturation of chondrocytes in both POC and SOC formation, and the regulatory mechanisms are similar. The deletion of *Phd2* stimulates HIF signaling, including *Hif-1α and Hif-2α*, HIF downstream targets, *Vegfa and Vegfb,* and increased bone mass [29,85]. At the stage of vascular invasion of the cartilage template, VEGF is expressed by the perichondrial osteolineage cells and hypertrophic chondrocytes to induce blood vessels to grow into the perichondrium [14]. The deletion of *Vegf* and lack of *Hif-1α* expression in COL2-expressing cells can lead to delayed blood vessel invasion or delayed POC formation [59]. It follows that the HIF-1/VEGF pathway may be involved in the POC formation. Some osteogenic transcription factors, including osterix and Runx2, may also be involved in regulating VEGF expression [86,87]. Runx2, which is upstream of osterix, can regulate the activity of VEGF in the osteolineage cells. Additionally, researchers observed that no blood vessels grew in the POC if Runx2 was deficient in mice [86,88]. At the stage of vascular invasion and the formation of the SOC, HIF signaling keeps the hypoxic chondrocytes alive. It regulates the expression of VEGF to induce vascularization in epiphyseal chondrocytes [89]. Maes et al. found that chondrocyte apoptosis could be reduced by overexpression of VEGF in HIF-1α-deficient mice [59]. Their study suggested that VEGF may be a downstream effector of HIF-1α to support the survival of chondrocytes. In addition, hypertrophic chondrocytes secrete VEGF to facilitate the formation of osteoclasts and play a crucial role in the entry of osteoclasts into the cartilaginous template [90]. Furthermore, during bone regeneration, hypertrophic chondrocyte-derived VEGF promotes the migration and differentiation of monocytes to osteoclasts [91].

During adult life, osteoblasts can express VEGF, which usually regulates endothelial migration, proliferation, and vessel permeability in a paracrine manner, and acts on pericytes and osteoclasts [6]. Additionally, osteoblasts can express all VEGF receptors, including VEGFR-1, VEGFR-2, and neuropilin [92]. Zelzer and Olsen concluded that VEGF might affect osteoblast biology in the following ways [93]. First of all, VEGF, expressed by osteoblasts, could induce angiogenesis to couple angiogenesis to bone formation. Secondly, VEGF directly regulates the differentiation and activity of osteoblastic in an autocrine manner. Thirdly, VEGF could act as a runner for bilateral regulation to induce the expression of cytokines to regulate osteoblastic activity in turn [94]. For example, human VEGF165 protein induces migration and alkaline phosphatase activity through binding to osteoblasts [95]. In addition, exogenous VEGF stimulates the mineralization of osteoblast cultures. However, it is reported that osteoblasts can produce Cxcl9, which prevents VEGF from binding to receptors on the surface of ECs and osteoblasts, holding back angiogenesis and osteogenesis. Additionally, the molecular mechanism involved remains to be further explored [96] (Figure 2D).

HIF-2α has been shown to be a critical regulator of endochondral ossification, enhancing promoter activities of COL10A1, MMP13, and VEGFA [49]. However, HIF-2 has recently been reported to regulate bone formation negatively. In particular, the differentiation of osteoblasts and osteoclasts during endochondral osteogenesis is regulated by HIF-2 independent of VEGF. On the one hand, HIF-2 regulates Sox9, which restricts Runx2 and Sp7, important osteoblastic differentiation regulators, which have an inhibitory effect on osteoblastic differentiation. On the other hand, HIF-2 regulates Twist2 to inhibit Runx2 and osteocalcin expression, which inhibits the mineralization of osteoblasts and reduces bone mass. In addition, Twist2 is also regulated by HIF-1 and has a similar negative regulatory effect on osteogenesis. In terms of osteoclasts, HIF-2 has been observed to promote the maturation and differentiation of osteoclasts, but it may not enhance the absorptive capacity of osteoclasts. In addition, HIF-2 also mediates the crosstalk between osteoblasts and osteoclasts [44,46]. Loss of specific HIF-2α in osteoclasts and osteoblasts can affect the activity of the other and ultimately increase bone mass [44]. More research is needed on the role of HIF-2 in bone formation (Figure 2E).

### 3.4. Skeletal Repair

The process of fracture healing is similar to several stages of endochondral bone formation, in which angiogenesis is necessary, and it can be divided into three phases: the inflammatory or initial phase of healing, the soft callus or intermediate stage, and the hard callus stage [97]. Additionally, the process is regulated by complicated interactions between the numerous cell types found in bone, primarily BMSCs, osteoblasts, osteoclasts, and osteocytes. After the fracture, vessels are damaged, and hematomas form, which limits blood perfusion and causes regional hypoxia (0.8–3% pO_2_). The lack of oxygen and nutrients upregulates HIFs and stabilizes HIF expression that up-regulates downstream VEGF expression [59,98]. In the fractured callus, varied cells can secrete VEGF, such as inflammatory cells, mesenchyme, osteoblasts, and hypertrophic chondrocytes [33]. Blood vessels not only form a local circulation to obtain nutrients and oxygen in the newly formed bone area but also directly promote bone formation. The endovascular blood sinus is the initial site of osteogenesis, and sufficient nutrients, oxygen, and minerals directly promote the formation and mineralization of an osteogenic matrix. In addition, VEGF also up-regulates downstream placental growth factor (PIGF). PlGF is a growth factor that belongs to the VEGF family, expressed by osteogenic cells, macrophages, and osteoclasts in the bone and marrow environment. After the fracture, PIGF is secreted in large amounts. On the one hand, it can recruit inflammatory cells to remove cellular debris, and on the other hand, it can promote angiogenesis and stimulate the proliferation and differentiation of periosteal cells into chondrocytes, secreting a large amount of cartilage matrix [99]. PlGF is also involved in the transformation of the cartilage matrix and regulates osteoclast formation during the formation of soft and hard calluses. Next, the hypertrophic chondrocytes undergo apoptosis, and the new osteoclasts are recruited to the fracture site. The damaged bone is then replaced by woven bone, and bone remodeling is performed [100]. The HIF-1–VEGF–PlGF pathway plays a significant role in fracture repair. HIF-2 also plays a role in fracture healing by stimulating angiogenesis. During defect repair, osteogenic precursor cells and blood vessels invade the bone defect area together to promote the formation of new bone [33].

Indeed, HIF-1 promotes angiogenesis and bone healing by regulating VEGF after fracture, which has been demonstrated in recent research. Zhang et al. have confirmed that HIF-1α is involved in the uMSC-Exos-induced VEGF expression and promotes fracture repair [101]. A recent study has shown that HIF-1α in chondrocytes in the calluses plays a role in bone healing. Partial HIF-1α deficiency in chondrocytes promotes chondrogenesis and affects the formation of vessels, osteoblasts, and osteoclasts during repair and remodeling, contributing to the bone-healing process [102]. Ding et al. observed that dimethyloxalylglycine (DMOG) could up-regulate the expression of HIF-1α to enhance the angiogenic activity of bone mesenchymal stem cells (BMSCs), which promote bone healing capacity [103]. Hypoxia-preconditioned BMSCs with the combined treatment of 1% O_2_ and DMOG showed up-regulated HIF-1α, enhanced VEGF expression and angiogenesis and osteogenesis in vivo and in vitro, promoting bone healing processes in geriatric individuals [104]. In rats treated with DFO during distraction osteogenesis, HIF-1α and VEGF expression increased in vivo, which contributed to bone regeneration, including by accelerating bone formation and remodeling [105]. HIFs may also control other signaling pathways independent of angiogenesis during bone healing, which requires further exploration (Figure 2F).

## 4. HIF/EPO Pathway

Under hypoxic conditions, EPO is also modulated by HIFs to increase production in the kidney and liver [55,106]. The PHD2: HIF-2α: EPO axis has been reported to be involved in the regulation of bone homeostasis [21]. Under hypoxic conditions, renal EPO is entirely induced by HIF-2, and the EPO enhancer possesses a hypoxia response element that can be activated by HIF-2 [42,107]. Erythropoietin (EPO) is a 30.4 kD circulating glycoprotein hormone. The kidney is the main organ that produces EPO, distributed in the blood circulatory system [108,109]. It has been discovered that EPO can bind to its receptor (EPOR) to promote erythropoiesis in the bone marrow. During embryogenesis, EPOR is expressed in the vasculature and endothelial progenitor cells, which act as the precursor of vascular endothelial cells and are stimulated to proliferate and differentiate [110,111]. It is reported that EPO also regulates the angiogenesis response in vivo [112]. During embryogenesis, angiogenesis starting at E10.5 would be affected if EPO and EPOR were inhibited in the vascular system. Studies have found that there may be a relationship between the regulation of erythropoiesis and bone homeostasis [113]. HIF-2 plays a major part in the regulation of EPO in both infant and adult stages. The role of HIF signaling regulation EPO to regulate bone homeostasis is reflected in the following aspects.

### 4.1. Bone Angiogenesis

Under hypoxia, EPO is particularly crucial for ischemic stress to increase tissue oxygen delivery by increasing the production of red blood cells in bone [114]. When tissues are hypoxic, HIF-2 stimulates EPO production in the kidney and liver to maintain normal serum EPO levels. In addition, it has been found that in patients with chronic mountain sickness, the local HIF-2α/EPO pathway in the bone marrow was involved in erythropoiesis and bone marrow microangiogenesis [115]. The study of Landau et al. characterized the (renal) HIF- (renal) EPO- (bone marrow) EPOR axis according to an animal experiment of anemic juvenile CKD rats, where the cells were deprived of oxygen [116]. EPO promotes the expression of Ang-1 that regulates endothelial cells by binding to its receptor, tyrosine kinase receptor 2(Tie2), suggesting that Ang-1 is one of the downstream molecules of EPO that regulates angiogenesis. Meanwhile, Tie2 in ECs may be cross-regulated in the EPOR signaling pathway because STAT3 and/or STAT5 are involved in regulating biological effects mediated by EPO. Under the condition of hypoxia, the binding of EPO to EPOR in the peripheral circulation generates downstream phosphorylation of the Janus kinase (JAK)/signal transducer and activator of transcription (STAT) signal transduction cascades in cells [117]. Wan et al. observed that EPO and VEGF stimulated endothelial sprouting in similar ways, suggesting that both factors may play a partially overlapping role in angiogenesis [118]. Therefore, it is not surprising that VEGF and EPO are often co-induced when tissue is anoxic, such as in tumors and trauma. However, VEGF is regulated by HIF-1 and HIF-2, while EPO is stimulated by HIF-2 [78]. Furthermore, Greenwald et al. have found that VEGF can enhance EPO expression of independently of hypoxia [119] (Figure 2A).

### 4.2. Endochondral Bone Formation

Some researchers reported that HIF signaling promotes EPO expression in osteoblasts and increases the trabecular bone volume. Rankin et al. demonstrated that osteoblastic PHD/VHL/HIF signaling was involved in hematopoiesis and was relevant to the proliferation of hematopoietic stem cells and erythroid progenitors [120]. Additionally, we hypothesize the coupling between osteogenesis and hematopoiesis is induced by PHD/VHL/HIF signaling in osteoblasts. Under the condition of hypoxia, the HIF-2/EPO pathway is activated to regulate the activity and function of cells and organs in response to hypoxia [121]. Concretely, this is mainly related to the following several pathways.

**Figure 3:**
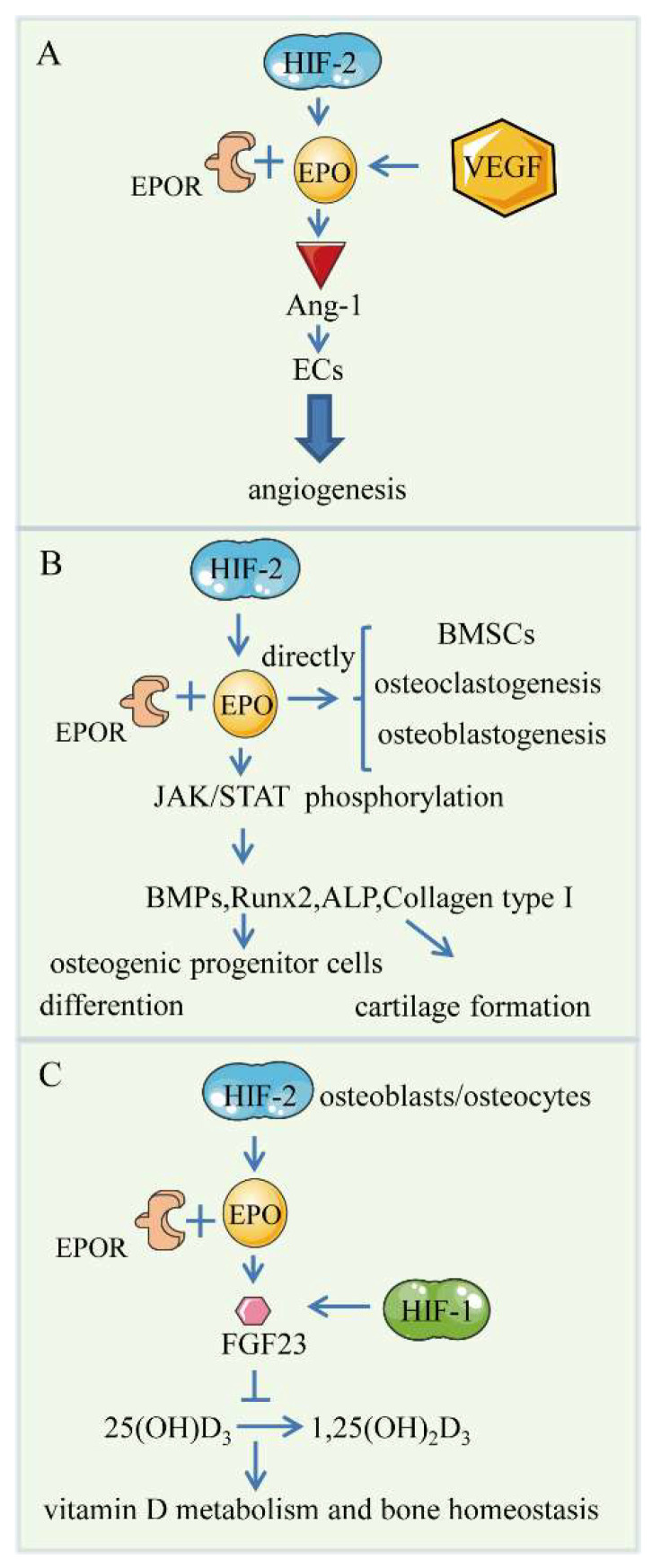
HIFs/EPO pathway. (**A**) Bone angiogenesis. HIF-2 can increase EPO production to up-regulate Ang-1 to promote angiogenesis. VEGF is a potent EPO inducer. (**B**) EPO phosphorylates the JAK/STAT signal transduction cascade in cells and induces differentiation of osteoblast progenitors and chondrogenesis. EPO can directly promote the differentiation of BMSCs and osteoclastogenesis and osteoblastogenesis. (**C**) HIF-2/EPO/FGF23 pathway. HIF-2 induces the production of EPO to regulate the regulation of calcium and phosphate metabolism on bone mineralization. FGF23 is also directly regulated by HIF-1.

The first pathway is the JAK/STAT signaling pathway. The binding of EPO to the preformed homodimer transmembrane receptor (EPOR) activates downstream JAK/STAT signal transduction cascades. Both osteoblasts and osteoclasts express EPOR for remodeling the bone, and BMSCs are also regulated by EPO [114]. In a study in vitro, the addition of EPO promoted phosphorylation of JAK2 and STAT3 proteins in osteoblasts, confirming that osteoblasts are regulated by EPO [122,123]. Next, BMPs, most notably BMP2 and BMP6, Runx2, ALP, and Collagen type I, are produced in quantity, which is associated with the differentiation of osteogenic progenitor cells. Meanwhile, BMP interacts with its receptors, BMPRs, to promote cartilage formation, which is vital to bone remodeling, particularly trabecular bone [123]. In addition, Shiozawa et al. reported that EPO also could act directly on BMSCs to induce an osteoblastic phenotype [117,124]. EPO can even stimulate the differentiation of mesenchymal cells in vitro, and promote bone formation in vivo. To be specific, EPO stimulates osteoclastogenesis first, followed by osteoblastogenesis. Moreover, EPO induces osteoclastogenesis but does not affect osteoclast function. In addition, it has been reported that EPO might also activate the ERK1/2 signaling pathway to stimulate BMSCs to differentiate into osteoblasts [125]. It follows that EPO regulates the differentiation of osteoblasts and osteoclasts and promotes bone formation in both direct and indirect ways, including certain signaling pathways [126] (Figure 3B).

The second pathway is the HIF-2/EPO/FGF23 pathway. Studies have indicated that HIF signaling up-regulates downstream EPO to control the transcription and translation of fibroblast growth factor 23 (FGF23), or directly regulate FGF23 in osteogenic cells [127,128,129]. As a hormone, FGF23, produced by osteoblasts and osteocytes, can regulate phosphate homeostasis and counter-regulate 1,25-dihydroxy vitamin D_3_(l,25(OH)_2_D_3_) [130]. FGF23 curtails the formation of l,25(OH)_2_D_3_ and accelerates its degradation to adjust the regulation of calcium and phosphate metabolism to the mineralization of bone [130]. Rats treated with high doses of l,25(OH)_2_D_3_ have been shown to have impaired bone mineralization [131]. On the other hand, a study has suggested that HIF-1α could stimulate the abnormal production of FGF23 in tumor-induced osteomalacia(TIO) and proposed that HIF-1α could directly activate the expression of FGF23 in a pathological state [132] (Figure 3C).

**Figure 4:**
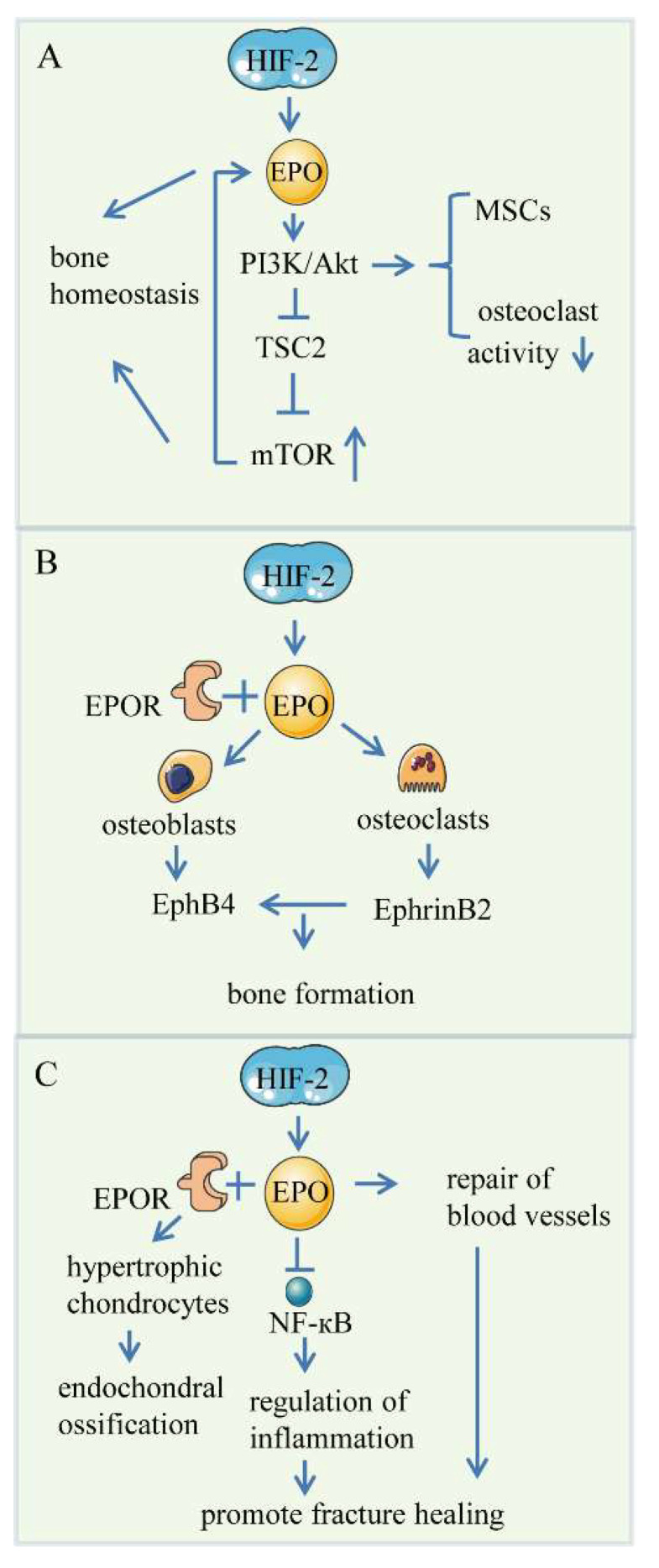
HIFs/EPO pathway. (**A**) PI3K/Akt signaling pathway. EPO stimulation activates PI3-kinase, and protein kinase B (PKB)/protein kinase-B (Akt) is activated downstream of PI3-kinase. Activated Akt phosphorylates decrease the ability of TSC2 to inhibit mTOR, resulting in the activation of mTOR, which mediates bone. EPO directly promotes the differentiation of MSCs into osteoblasts via the PI3K/AKT signal pathway and decreases osteoclast activity at the same time. (**B**) EphrinB2/EphB4 signaling pathway. EPO induces enhanced expression of EphB4 by osteoblasts, which, when activated by EphrinB2 expressed by osteoclasts, leads to enhanced osteoblast activity, increasing bone formation. (**C**) Skeletal repair. EPO lowers NF-κB expression and regulates inflammation, promoting fracture healing. At the same time, EPCs migrate to ischaemic areas or defect sites to improve the repair of blood vessels. EPO interacts with EPOR-expressed hypertrophic chondrocytes to promote endochondral ossification.

The third pathway is the PI3K/Akt signaling pathway. EPO recruits the p85 regulatory subunit to EPO-R Y479 and then activates phosphatidylinositol 3 kinase (PI3-kinase) signaling, which in turn phosphorylates and activates downstream protein kinase B (PKB)/protein kinase-B(Akt) [133]. Activated Akt activates the mammalian target of rapamycin(mTOR) by protecting mTOR from inhibition by tuberous sclerosis complex 2 (TSC2). Kim et al. reported the effects of mTOR signaling on EPO-mediated osteoblastogenesis and osteoclastogenesis, and they found that the inhibition of mTOR in human bone marrow stromal cells (hBMSCs) and bone-marrow-derived stromal cells affected the expression of EPO-dependent and EPO-independent osteoblastic phenotypes [134]. Additionally, the inhibition of mTOR also inhibited EPO-dependent and EPO-independent osteoclastogenesis in mouse bone marrow mononuclear cells and Raw264.7 cells [134]. They also found that EPO increased osteoclast numbers and decreased resorption activity, which is independent of mTOR. Hiram-Bab et al. also suggested that EPO also affected preosteoclasts and activated the Jak2 and PI3K pathways to promote osteoclastogenesis in isolated cultures [135]. In addition, the PI3K/AKT signal pathway is involved in the EPO stimulation of mesenchymal stem cells (MSCs) into osteoblasts (Figure 4A).

Another pathway is the EphrinB2/EphB4 signaling pathway. Li et al. studied how EPO affects the communication between osteoblasts and osteoclasts via the downstream EphrinB2/EphB4 signaling pathway to regulate bone formation [136]. EPO can increase EphrinB2-expressing osteoclasts but lower their ability to resorb bone. EphrinB2 significantly promoted osteoblastic differentiation of bone-marrow-derived stromal cells through the EphrinB2/EphB4 signaling pathway. It follows that EPO contributes to the bone formation by promoting osteoblastic phenotypes directly or indirectly by activating the EphrinB2/EphB4 signaling pathway (Figure 4B).

Furthermore, Wang et al. found that EPO may activate the p38 mitogen-activated protein kinase (MAPK) pathway to induce osteogenic differentiation of human periodontal ligament tissue-derived mesenchymal stem cells (hPDLSCs) and periodontitis mesenchymal stem cells (pPDLSCs) [137]. EPO has also been found to facilitate the osteogenic differentiation of hPDLSCs through activation of the Wnt/β-catenin signaling pathway [138].

EPO activates downstream molecules to regulate bone mass through the above signaling pathways in cells. Therefore, we theorize that, when activated by HIF-2 in a hypoxia state, EPO could further activate downstream intracellular signaling molecules to regulate bone mass.

### 4.3. Skeletal Repair

EPO-EPOR signaling functions in fracture healing by accelerating early endochondral ossification and enhancing mechanical strength [139]. Studies have shown that local application of EPO in long bone defects can effectively promote bone repair, and local and systemic application can also play roles to some extent in animal experiments [140]. Specifically, EPO could accelerate endochondral ossification by binding to EPOR expressed by hypertrophic chondrocytes and regulate the differentiation of BMSCs into osteoblasts [118]. In addition, when fractures occurred, the expression of NF-κB was significantly reduced in the EPO-treated mice, which promoted fracture healing [124,141]. NF-κB, a pivotal regulator of inflammation, induces excessive inflammation that harms bone formation [142]. Meanwhile, blood vessels are accelerated to repair the bone defect sites as EPCs migrate, proliferate, and differentiate [4] (Figure 4C).

### 4.4. Controversial Role of EPO in Bone Formation

However, EPO has also been shown to regulate bone mass negatively in other studies, and its principal function is bone resorption. Suresh et al. found that chronic elevated EPO levels reduced bone mass in mice [114]. Concretely, they found that elevated EPO inhibited BMP2-induced bone formation, and in BMSCs, high EPO level reduced their differentiation into osteoblasts and adipocytes. They reported that abnormal endogenous erythropoietin negatively affected the differentiation of BMSCs and that elevated exogenous EPO administration reduced trabecular bone in both male and female mice [114,123]. Oikonomidou et al. reported that chronically elevated circulating EPO can cause polycythemia and bone loss [143]. However, EPO has little effect on bone healing for the elderly, and this may be because of the gradual weakening of microcirculation [144]. Naamit et al. observed that high EPO-level treatment induces bone loss. They suggested that this might be due to the regulatory role of EPO on B cells. Specifically, on the one hand, EPO increases the expression of osteoclastogenic molecules (e.g., RANKL) on B cells; on the other hand, EPO promotes B cells to transdifferentiate into osteoclasts [145]. It is also probable that the exact role of EPO depends on the different experimental conditions [135].

The regulatory mechanism of EPO on bone in an anoxic environment is quite complex and not completely clear. Consequently, how EPO acts in regulating the skeletal homeostasis and regeneration under hypoxia is still a controversial topic. There are some differences in the published conclusions on the regulation of bone by EPO, possibly due to the different designs of experimental conditions, such as the animal models used and the dosage of drugs. Presently, we still encounter some problems with the role of EPO in osteogenesis and fracture repair mechanisms: for example, whether the regulation of bone by EPO is coupled to or secondary to hematopoiesis when regulating bone formation, what the optimal dose and time for EPO to promote bone formation in humans is, and under persistent hypoxia, whether EPO overexpression mediated by HIF-2 would affect osteogenesis. Therefore, the molecular mechanisms of EPO regulating bone homeostasis remain to be further explored.

## 5. Wnt Signaling Pathway

Studies found that hypoxia could affect the regulation of Wnt signaling on bone growth and homeostasis, indicating that HIF-1 may have a certain effect on regulating Wnt signaling [16]. The Wnt signaling pathway is a complex network of protein interactions that functions commonly in embryonic development and cancer, but is also involved in normal physiological processes in adulthood [146]. Specifically, it is involved in tissue homeostasis and diseases in various tissues [147]. The canonical Wnt pathway, also known as the β-catenin dependent pathway, is one of the three major Wnt signaling pathways. The others are the planar cell polarity pathway and the Wnt/Ca^2+^ pathway [148]. The canonical Wnt signaling pathway is the best-characterized and is closely involved in bone tissue engineering [147,148]. Animal studies have reported that canonical Wnt signaling could directly or indirectly regulate bone homeostasis. Specifically, the activation of Wnt signaling has a positive effect on bone mass [149].

### 5.1. Bone Angiogenesis

**Figure 5:**
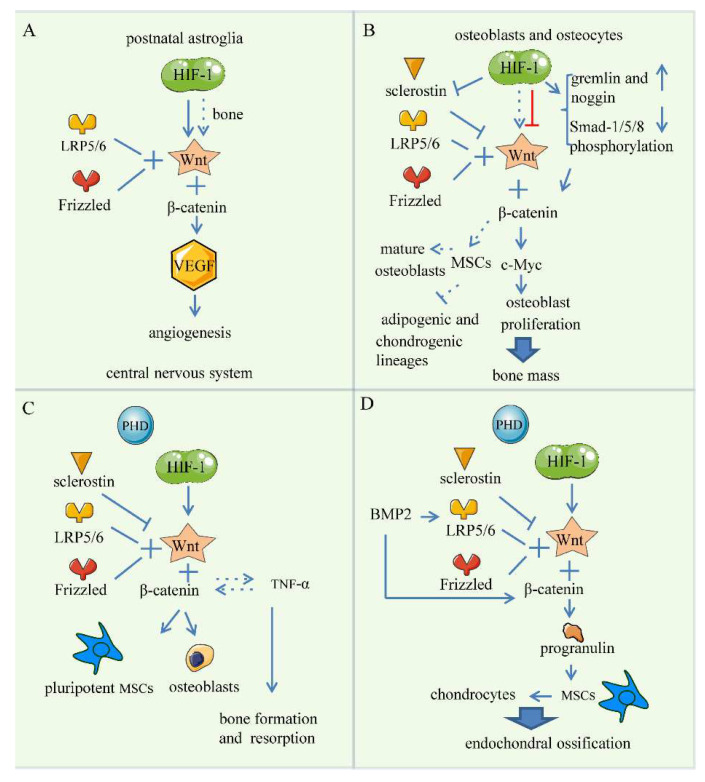
Wnt Signaling Pathway. (**A**) Bone angiogenesis. HIF-1 up-regulates Wnt7a/7b in oligodendrocyte precursor cells, and Wnt/β-catenin signaling regulates VEGF transcription and promotes vessel growth. (**B**) Endochondral bone formation. HIF-1 activates Wnt to increase the expression levels of c-Myc to promote the osteoblast proliferation. Sclerostin inhibits Wnt signaling. HIF-1 increases expression of the BMP antagonists and decreases Smad-1/5/8 phosphorylation to decrease sclerostin transcript in osteoblasts and osteocytes. β-catenin promotes the differentiation of MSCs into mature osteoblasts and inhibits the differentiation into adipogenic and chondrogenic lineages. HIF-1 is also found to inhibit Wnt signaling. (**C**) Skeletal repair. The interaction between the Wnt pathway and TNFα mediates inflammatory process and regulates the balance between skeletal bone formation and resorption. (**D**) Skeletal repair. Wnt pathways regulate the progranulin that promotes the differentiation of MSCs into chondrocytes as well as endochondral ossification. BMP2 increases the expression of LRP5 and stabilizes β-catenin promoting osteogenic differentiation.

Wnt pathways are indispensable in angiogenesis and vessel remodeling [150]. Yuen et al. observed that HIF-1α could bind to conserved HREs at the Wnt7a and Wnt7b loci, resulting in up-regulation of Wnt7a/7b in oligodendrocyte precursor cells [151]. In addition, Zhang et al. observed that HIF-1-activated Wnt signaling is partially involved in the regulation of angiogenesis by astroglia in the central nervous system (CNS), which reveals a glial cell-type-specific HIF-1/Wnt connection in the CNS [152]. It can be seen that HIF stabilization can activate cell-autonomous Wnt production in CNS. However, the role of HIF-1 in the direct regulation of the Wnt pathway to promote angiogenesis in bone has not been specifically reported in the literature. Wnt/β-catenin signaling has been shown to regulate the transcription of VEGF. Furthermore, active β-catenin and Wnt signaling can overexpress VEGF in adenomatous polyposis coli [79]. Therefore, we speculate that HIF-1 is associated with the Wnt pathway through the VEGF to some extent during bone angiogenesis and bone formation, which still needs to be further studied (Figure 5A).

### 5.2. Endochondral Bone Formation

Since both the Wnt signaling pathway and the HIF pathway have been shown to have profound effects on bone homeostasis, researchers investigated whether these pathways are related or interacted. Li et al. have confirmed that HIF-1 has an impact on activation on Wnt in osteoblasts, and ultimately promotes osteoblast proliferation by increasing c-Myc and other cytokines [36].

Sclerostin is transcribed and translated by the *Sost* gene and acts against Wnt/β-catenin. It competes with Wnt by binding to LRP5/6, inhibiting the transduction of Wnt signaling. It has been reported that sclerostin regulates the activity of some osteogenesis-related cells, such as increasing osteoblast apoptosis and reducing osteoprogenitor proliferation, which ultimately has a negative effect on bone mass [153]. When tissue is hypoxic, HIF-1 signaling reduces sclerostin expression, which enhances Wnt/β-catenin signaling in osteocytes to regulate bone mass. Specifically, HIF-1 deacetylates the *Sost* promoter to decrease sclerostin expression [154]. Stegen et al. observed that deleting *Phd2* in osteocytes also promotes deacetylation of the *Sost* promoter, thereby reducing the expression of sclerostin, which leads to increased expression of canonical Wnt signaling and bone mass [16,155]. Similarly, Genetos et al. reported that knockout of *Vhl* in osteocytes stabilized the expression of HIF-α isoforms to increase the expression of canonical Wnt signaling [156]. In addition, it has been reported that hypoxia increases the expression of Gremlin and Noggin and reduces phosphorylation of Smad-1/5/8, which lowers the expression of sclerostin and activates the Wnt signaling in osteoblasts and osteocytes [156]. Therefore, according to the fact that Wnt/β-catenin signaling is up-regulated in a low-oxygen environment, we hypothesize that HIF-1 can active Wnt signaling in osteoblasts and osteocytes to promote bone formation (Figure 5B).

However, HIF-1 inhibits the Wnt/β-catenin signaling pathway in osteoarthritis and lowers MMP13 to reduce catabolic activity [51]. At the same time, HIF-1α down-regulates the interaction between Runx2 and TCF-4/β-catenin and interrupts Wnt signals to reduce endochondral ossification. In terms of cartilage, hypoxia has a dual effect on chondrocytes: HIF-1 is activated and silences Runx2 and Wnt to accelerate matrix synthesis. In another aspect, when proinflammatory cytokines are present, HIF-2 is activated by hypoxia conditions. Next, VEGF and MMP13 are up-regulated, and aberrant collagen expression with extracellular matrix deterioration [155]. Zhou et al. demonstrated that HIF-1α could potentiate BMP2-induced cartilage formation with Sox-9 up-regulating [157]. Additionally, the role of the Wnt signaling pathway in the osteogenic differentiation of MSCs has been confirmed, and β-catenin can also suppress that MSCs differentiate into adipogenic and chondrogenic lineages [148,158] (Figure 5C).

### 5.3. Skeletal Repair

The canonical Wnt signaling pathway is indispensable in skeletal tissue regeneration and repair. β-catenin performs different functions at different stages of bone repair. In the early stage of repair, β-catenin in the callus modulates the relative amount of osteoblasts and chondrocytes arising from MSCs. During the late bone healing process, β-catenin acts to stimulate the production of the more osteoblastic matrix [159]. Wnt signaling also regulates bone immunity [148]. The Wnt pathway may interact with tumor necrosis factor (TNF)-α, an indispensable regulator of inflammation and immunity, to co-regulate the inflammatory process, which is quite complex. Except for TNFα, Wnt signaling also intersects with other signals regulating bone formation and repair. For example, in frontotemporal dementia, progranulin growth factor can be regulated by Wnt pathways. Additionally, progranulin is associated with stimulating the differentiation of MSCs into chondrocytes [160]. MicroRNAs have been the focus of recent research. They interact with WNT signaling to promote osteogenic differentiation of MSCs. For example, overexpression of MiR-26a, MiR-199b-5p, and MiR-346 increases nuclear β-catenin levels and activates the Wnt signaling pathway to enhance osteoblast differentiation. Mir-218 down-regulates the inhibitors of the Wnt signaling pathway, namely DKK2, sFRP2, and sclerostin, and activates the Wnt signaling pathway [161]. Furthermore, it is reported that Wnt signaling also mediates MicroRNAs to induce osteogenic differentiation. In addition, BMP2 can promote the expression of LRP5 and down-regulate β-Trcp to maintain the stability of β-catenin to facilitate osteogenic differentiation [162]. Kim et al. demonstrated that constitutive activation of Wnt signaling affected the differentiation of osteoblast and reduced bone mineralization [163]. Thus, Bao et al. hypothesize that an opportune level of Wnt/β-catenin signaling contributes to better healing at the late stage of fracture healing [159] (Figure 5D).

Nevertheless, in another study, Chen et al. observed that HIF-1α cooperating with osterix (Osx) activates the expression of *Sost* to inhibit the Wnt pathway, and the activity of osteoblast is suppressed [164,165]. Besides, in embryonic stem cells, HIF-1α could activate Wnt signaling by up-regulating lymphoid enhancer-binding factor 1(LEF1) and transcription factor 1(TCF1). In contrast, in colorectal cancer cells, HIF-1α inhibits canonical Wnt signaling by blocking the TCF4–β-catenin interaction and transcriptional activity [166]. Based on the above research, we conclude that hypoxia/HIF-1 has both positive and negative effects on the regulation of Wnt signaling, which may vary with the type of cells.

## 6. SHIP-1

It has been reported that Src homology 2-domain-containing Inositol 5′-Phosphatase-1 (SHIP-1) and HIFs interact to regulate the proliferation of MSCs. SHIP was originally identified as a tyrosine-phosphorylated protein that is activated by various cytokines and growth factors in blood cells. SHIP motifs are composed of 1190 amino acids and are important for protein–protein interactions. The 145-kD protein can be named SHIP, SHIP-1, or SHIPα [167]. SHIP can regulate specific myeloid cells by regulating Phosphatidylinositol (3,4,5) trisphosphate (PIP_3_) (PI(3,4,5)P3) and subsequently activating Akt/PKB to keep a growth balance. As a center of the cell signaling pathway, phosphatidylinositol-3-kinase (PI3K) has a wide range of effects on cellular physiology. PI3K interacts with phosphatidylinositol (4,5)-bisphosphate (PI(4,5)P2) and the secondary messenger PI(3,4,5)P3 forms. PI (3,4,5) P3 binds to the SH2 domain of SHIP and recruits SHIP to the cytomembrane [168]. Additionally, it can hydrolyze PI(3,4,5)P3 to PI(3,4)P2, through which SHIP limits membrane recruitment and activation of Akt to modulate the activity of PI3K. SHIP includes two main subtypes, SHIP-1 and SHIP-2, related to the PI3K pathway. The cells of the hematopoietic lineage, osteoblasts, and mesenchymal stem cells can express SHIP-1. However, SHIP-2 is expressed in almost all cell and tissue types.

### 6.1. The Regulation in Osteoblasts and in MSCs

**Figure 6:**
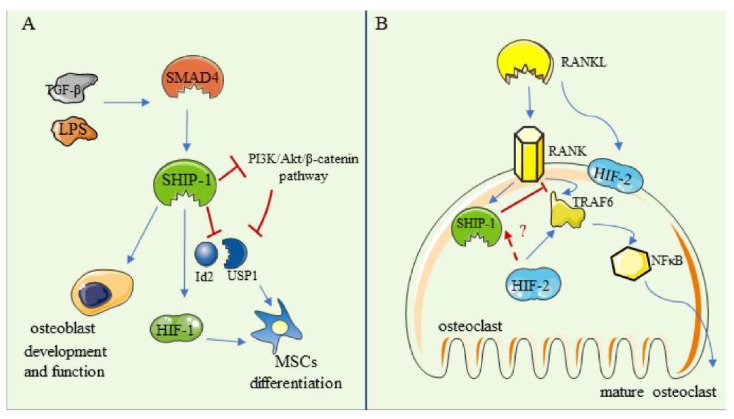
SHIP-1 Signaling. (**A**)The regulation in osteoblasts and MSCs. SHIP is involved in the development and function of osteoblasts directly. SHIP-1 stabilizes the expression of HIF-1 and regulates the differentiation of MSCs. (**B**) The regulation in osteoclasts. SHIP-1, downstream of RANK, negatively regulates the activity of osteoclasts. HIF-2α can directly up-regulate TRAF6 and increase osteoclastogenesis.

In the biology of bone, Iyer et al. reported that the signals downstream of SHIP-1 are involved in the regulation of biological activities [169]. Hazen et al. found that alkaline phosphatase (ALP) was expressed less in osteoblasts, conditionally knocking out *Ship1* in vitro [170]. This suggests that SHIP may be involved in the development and function of osteoblasts directly, such as bone mineralization. Iyer et al. also expounded on the new role of SHIP-1 in regulating the differentiation of MSCs [171]. They speculated that SHIP-1 could be regulated by SMAD4 to mediate osteolineage commitment because transforming growth factor-β (TGF-β) and bacterial lipopolysaccharide induce SMAD family transcription factors to stimulate the secretion of SHIP-1. SHIP-1 can promote osteoblast development by decreasing the inhibitor of differentiation2 (Id2) levels in MSCs and sequestering USP1 from interacting with Id2. In particular, Iyer et al. found that SHIP-1 inhibited Id2 by restricting the activation of the PI3K/Akt/β-catenin pathway, which is an Id2 activation-dependent pathway [172]. Therefore, SMAD4 induces SHIP-1 in MSCs and represses USP1/Id2, which promotes the differentiation of MSCs [169] (Figure 6A).

SHIP-1 performs a significant part in HIF-1 signaling in the growth and differentiation of MSCs. In the PDGFRα + Sca-1 + (PαS) MSC, the deficiency of SHIP-1 resulted in impaired HIF-1α stabilization. [38] Further, Takeshita et al. reported that when SHIP-1 is deficient in cells, the activation of Akt and extracellular-signal-regulated kinase (Erk) are also inhibited, which are generally activated under the condition of hypoxia, and when the level of HIF-1α protein is decreased, the proliferation of SHIP-1KO PαS MSC is also inhibited, and the phosphorylation levels of Akt and Erk are decreased, which promotes the hypertrophic differentiation of MSCs [38,172].

### 6.2. The Regulation in Osteoclasts

Takeshita et al. reported that SHIP had a negative regulatory effect on osteoclast activity, and severe osteoporosis would occur without this enzyme [172]. This is because if SHIP is lacking, the sensitivity of marrow macrophages to the macrophage-colony stimulating factor (M-CSF) and the receptor activator of nuclear factor-κB ligand (RANKL) will be enhanced, and hyperresorptive osteoclasts increase and lead to osteoporosis. In terms of differentiation and function of osteoclasts, SHIP-1 is downstream of RANK. When HIF-2α is stable, HIF-2α can directly up-regulate TRAF6, an adapter of RANK, thereby activating Nfatc1 and increasing osteoclastogenesis. HIF-2 may also promote osteoclast differentiation by directly binding to the RANKL promoter [44,45]. Therefore, we hypothesize that HIF-2 might interfere with SHIP activity and up-regulate TRAF6, thereby regulating osteoclast differentiation and activity. As a result, it affects bone formation, including bone quantity, quality, density, and strength. This remains to be confirmed by extensive experimental studies (Figure 6B).

Hence, SHIP-1 is indispensable for regulating hypoxia signaling in the growth of MSCs and bone homeostasis. However, small molecule phosphatase modulators, including SHIP inhibitors, are now developing. Researchers want to treat certain diseases by regulating and controlling the activity of SHIP. SHIP can modulate the PI (3, 4, 5) P3 to influence the downstream signaling of the PI3K pathway, which is an essential signaling pathway in the physiological and pathological activities of mammalian cells. The signals received by a receptor on the cell membrane are transferred to the nucleus. These signals are involved in cell division, survival, and other cellular functions by modulating transcription and protein synthesis. Therefore, SHIP activity might be associated with the occurrence and treatment of a disease. Additionally, there are studies that indicate that small molecule SHIP inhibitors play a profound part in the treatment of some diseases, such as cancer, diabetes, obesity, and Alzheimer’s disease [168,173]. At the same time, inhibition of SHIP-1 activity may also affect the stability of HIFs and the response of systemic cells to hypoxia. Therefore, the negative effects of SHIP inhibitors on bone homeostasis should be more carefully considered when using SHIP inhibitors.

## 7. Conclusions

During bone development, disease, and regeneration, the HIF signaling pathway performs an indispensable role in the adaptation of limb bud mesenchyme and osteocytes to physiological and pathological hypoxia in the bone marrow microenvironment and promotes their growth and differentiation. It mediates the cell survival and angiogenesis processes necessary for bone tissue engineering [174]. Hypoxia can stimulate the expression of HIF-αs when oxygen levels drop to <5%. To date, HIFs have been found to regulate 200 target genes to control a variety of biological processes [46,175]. As a classic downstream target of the HIFs pathway, VEGF can regulate angiogenesis and promote skeletal development and bone homeostasis. HIF-2 actives EPO in the kidney and liver to regulate erythropoiesis homeostasis and thus affect bone homeostasis. In addition, HIF-1α can activate Wnt signaling in osteoblasts and osteocytes, regulating bone mass. SHIP-1 is also a factor that affects the expression of HIF-1α through the PI3K-AKT signaling pathway to regulate osteoblasts and osteoclasts. Researchers found that osteoclasts and osteoblasts differ in their sensitivity to HIF. Osteoclasts are more susceptive to HIF suppression, while HIF activation is more robust in osteoblasts [176]. Nevertheless, there are some studies suggesting that hypoxia might inhibit bone formation. Zhang et al. observed that hypoxia suppressed osteogenesis of BMSCs via ERK1/2 and p38-mitogen-activated protein kinase (p38-MAPK) signaling pathways when BMSCs were exposed to 2% O_2_ [177]. Additionally, with regard to HIF-1α, Liu et al. observed that HIF-1α and TGF-β1 could synergistically inhibit the osteogenesis of PDLSCs [178]. The distinctions in the effects of hypoxia on the behaviors of BMSCs may be related to the differences in O_2_ concentration and exposure time among different studies [177]. In osteogenesis and bone repair, both HIF-1 and HIF-2 increase angiogenesis, and HIF-1 has a positive effect on osteogenesis. However, HIF-2 has a partially negative impact on the regulation of bone homeostasis by inhibiting the proliferation and differentiation of osteoblasts. HIF-1 couples osteogenesis and angiogenesis, and promotes cellular metabolic transformation for survival, while HIF-2 may play a regulatory role in bone remodeling by regulating the activity and function of osteoblasts and osteoclasts [44,46].

Studies have suggested that hypoxia could induce an osteogenic–angiogenic response, which is a potential therapeutic direction for bone regeneration. The mechanism by which HIF regulates bone metabolism is quite complex. In tumors, HIF-1 and HIF-2 are also involved in angiogenesis, and tumor development and metastasis, especially HIF-2 in solid tumors. Therefore, more attention is required to investigate further the complicated molecular mechanisms involved in the regulation of HIFs on cell activities and biological effects under the condition of hypoxia. We need to regulate HIFs to be beneficial to bone regeneration while not promoting tumors. In particular, the study of bone tissue engineering related to the HIF signaling pathway will be an exciting and meaningful research direction.

## Figures and Tables

**Table 1:** HIF-1 functions through regulating different signals in different animal models or cells.

HIFs	Mouse Models /Cells	Signaling Pathway	Effects	Ref.
HIF-1	EC-specific loss-of-function mice (*Hif1a*^iΔEC^)	HIF-1/VEGF	An increased number of type H vessels and enhanced endochondral angiogenesis and osteogenesis	[3,33]
	Mature osteoblasts	HIF-1/VEGF	Contribute to the coordination of vascularization, ossification and matrix resorption in endochondral bone development	[16,24]
	The mouse model of hindlimb ischemia	HIF-1/VEGF	HIF-1 activation in myeloid cells promotes angiogenesis	[34]
	HIF-1α-deficient embryos	HIF-1/EPO	Affect embryonic development	[35]
	rat calvaria bone defect model	HIF-1/EPO	Promote osteogenesis and accelerate bone repair	[4]
	MC3T3-E1	HIF-1/Wnt	Promote osteoblast proliferation	[36]
	BMSCs in osteonecrosis of the femoral head	HIF-1/β-Catenin	Reduce cellular apoptosis, lower empty lacunae rate, enhance bone formation, and stronger trabecular bone	[37]
	PDGFRα + Sca-1+(PαS) MSC	SHIP-1	SHIP-1 maintains the stable expression of HIF-1α in Pαs MSC under hypoxia, and reduced the expression of HIF-1α inhibits the proliferation of SHIP- 1KOPαs MSC	[38]
	periapical lesions in mice	HIF-1/NF-κB	Attenuate periapical bone loss, inhibit osteoclasts	[39]
	MSCs	HIF-1/CXCR4 and CXCR7	Promote MSCs migration and survival capacity	[40]
	10-wk-old osteoclast-specific HIF-1α conditional knockout mice	HIF-1/AMPK	Maintain osteoclast-induced resorption of calcified cartilage matrix	[41]

**Table 2:** HIF-2 functions through regulating different signals in different animal models or cells.

HIFs	Mouse Models /Cells	Signaling Pathway	Effects	Ref.
HIF-2	Mature osteoblasts	HIF-2/VEGF	Contribute to the coordination of vascularization, ossification and matrix resorption in endochondral bone development	[54]
	HIF-2α-ablated mice	HIF-2/EPO	Affect adult EPO synthesis	[55]
	N1511 mouse chondrocytes	HIF-2/Fas	Mediate chondrocyte apoptosis and regulates autophagy in maturing chondrocytes	[56,57]
	Human CD14+ monocytes	--	Modulate osteoclast differentiation and formation	[58]
	Male mice	HIF-2/*p16* and *p21*	Act as a senescence-related intrinsic factor in age-related dysfunction of bone homeostasis	[47]
	Murine experimental OA models	NF-κB-HIF-2α pathway	Promote OA development	[48]

## Data Availability

Not applicable.
